# Juvenile systemic lupus erythematosus: a retrospective study in a single center over a 13 years period

**DOI:** 10.1186/1546-0096-9-S1-P266

**Published:** 2011-09-14

**Authors:** N Conejo, O Porras

**Affiliations:** 1 National ChildrenÂ´s Hospital “Dr. Carlos Sáenz Herrera”, Department of Pediatric Immunology and Rheumatology, San José, Costa Rica

## Aim

To evaluate a group of children diagnosed as juvenile systemic lupus erythematosus (JSLE) in a single center over a 13-year period.

## Methods

JSLE patients, age ≤ 18 years, were included. Clinical presentation, laboratory parameters and therapeutic approaches were analyzed at diagnosis. Outcome, response and adverse events to treatment were analyzed during the follow up period.

## Results

74 cases were included, 78.3% females. The mean age at diagnosis was 9.9 years, 54% were children of age 6-10 years and 35.1% were of age 11-14 years. The most frequent pre-diagnosis manifestations were fever, arthralgias, asthenia, adinamia, weight loss, headaches and anorexia. The four most frequent classification criteria were a positive ANA (91.7%), positive immunoserology (95.9%), cytopenia (75.7%) and nephritis (58.1%). The main complications were associated to nephritis (58.1%), central nervous system (40.5%) and infection (24.3%). Seventy cases had flares, in relation with nephritis (35.7%), malar rash (27,1%), arthritis (20%), vasculitis (15.7%), serositis (11.4%) and cytopenias (10%). Fifteen children died (20.3%), due to septic shock, chronic renal insufficiency, autoimmune hepatitis, cerebral infarct, lung hemorrhage, hypertensive encephalitis, antiphospholipid syndrome and renal thrombosis. Death occurs a mean of 2.5 years (9 days to 7.9 years) after diagnosis.

## Conclusions

A group of 74 children with JSLE were evaluated, most of them female. Half of the cases developed nephritis. A fifth of the cases died during the study period, mainly from infection. The analysis of the studied group allows us to obtain clinical information crucial to define diagnosis and treatment guidelines and develop medical education material.

